# Validation of an anti-α-Gal IgE fluoroenzyme-immunoassay for the screening of patients at risk of severe anaphylaxis to cetuximab

**DOI:** 10.1186/s12885-023-10501-5

**Published:** 2023-01-09

**Authors:** Julien Serrier, Jean-Baptiste Davy, Benoît Dupont, Bénédicte Clarisse, Jean-Jacques Parienti, Gautier Petit, Kathy Khoy, Yann Ollivier, Radj Gervais, Delphine Mariotte, Brigitte Le Mauff

**Affiliations:** 1 Department of Immunology and Histocompatibility, CHU Caen, Caen, France; 2 University of Caen Normandy, Caen, France; 3 INSERM U1237, Physiopathology and Imaging of Neurological Disorders, Caen, France; 4 Department of Hepato-Gastroenterology and Nutrition, CHU Caen, Caen, France; 5 Clinical Research Department, Centre François Baclesse, Caen, France; 6 Department of Clinical Research and Biostatistics, CHU Caen, Caen, France; 7 University Center for Allergic Diseases (CUMA), CHU Caen, Caen, France; 8 Medical Oncology Department, Centre François Baclesse, Caen, France

**Keywords:** Anaphylaxis, Cetuximab, Galactose-alpha-1,3-galactose (alpha-Gal), Fluoroenzyme-immunoassay, Drug allergy

## Abstract

**Background:**

The link between immediate hypersensitivity reactions (HSR) following the first cetuximab infusion and the IgE sensitization against anti-galactose-α-1,3-galactose (α-Gal) is now well-established. An automated Fluoroenzyme-Immunoassay (FEIA) is available and may facilitate the screening of patients with anti-α-Gal IgE before treatment.

**Methods:**

This study aimed to evaluate its performances as compared to a previously validated anti-cetuximab IgE ELISA, using 185 samples from two previously studied cohorts.

**Results:**

Despite 21.1% of discrepancies between the two techniques, FEIA discriminated better positive patients and similarly negative ones with a ≥ 0.525 kU_A_/L threshold. Sensitivity was 87.5% for both tests, specificity was better for FEIA (96.3% vs ELISA: 82.1%). FEIA had a higher positive likelihood ratio (23.9 vs ELISA: 4.89) and a similar negative likelihood ratio (0.13 vs ELISA: 0.15). In our population, the risk of severe HSR following a positive test was higher with FEIA (56.7% vs ELISA: 19.6%) and similar following a negative test (0.7% vs ELISA: 0.8%).

**Conclusion:**

Although the predictive value of the IgE screening before cetuximab infusion remains discussed, this automated commercial test can identify high-risk patients and is suitable for routine use in laboratories. It could help avoiding cetuximab-induced HSR by a systematic anti-α-Gal IgE screening before treatment.

## Background

Cetuximab, a chimeric mouse/human monoclonal antibody, is an Epidermal Growth Factor Receptor (EGFR) inhibitor developed in the early 2000s for the treatment of patients with metastatic colorectal or head and neck cancer. The high frequency of immediate anaphylactic hypersensitivity reactions (HSR) following the first injection of this drug has led to the identification of IgE antibodies directed against galactose-alpha-1,3-galactose (α-Gal), an oligosaccharide epitope located on the variable domain of the heavy chain of cetuximab [1]. Even though IgE levels are not usually correlated with the severity of the reactions, very high concentrations of anti-α-Gal IgE have been found in sera of patients who underwent severe anaphylaxis with fatal outcome [2, 3]. Alpha-Gal, which is part of glycoproteins and glycolipids in all mammals except humans, apes, and old-world monkeys is an antigen frequently recognized by human IgM and IgG natural antibodies [4, 5]. Anti-α-Gal IgE have also been identified as responsible of anaphylactic reactions after ingestion of red meat (α-Gal syndrome) [6, 7]. Sensitization is generally assumed to be caused by tick bites. Accordingly, geographical disparities in the prevalence of anaphylactic reactions to cetuximab likely reflect differences in tick species distribution [8], with less than 1% of individuals in north-east of the United States exhibiting sensitization to cetuximab as compared to more than 20% in the south-eastern area [9]. In addition to tick exposure which is increasing worldwide, systemic administration of animal products (gelatins especially) has been identified as a cause of α-Gal sensitization [10].

In sensitized patients, immediate HSR can occur following the first cetuximab infusion, highlighting the importance of identifying high risk patients [11, 12]. Thus, an anti-cetuximab IgE screening has been suggested as a reliable strategy to identify those patients [13]. Moreover, we have validated an ELISA method in a prospective multicenter study for the screening of patients at high risk of immediate HSR before cetuximab infusion [11]. However, this test has some limitations, mainly related to the “home made” format and the absence of standardization.

An automated anti-α-Gal IgE Fluoroenzyme-Immuno Assay (FEIA) (ImmunoCap o215, Thermo-Fisher scientific) has been available since 2015 using bovine thyroglobulin, a protein extensively glycosylated with α-Gal [14]. This test is clinically validated to document the presence of specific anti-α-Gal IgE in patients with « delayed» meat allergy or allergic reactions to animal derived gelatins but not for anti-α-Gal IgE screening before cetuximab infusion [15, 16].

The aim of this study was to evaluate the diagnostic performances of this FEIA technique to detect α-Gal epitopes of cetuximab and to assess HSR risk using the two cohorts of patients previously tested with our validated ELISA method.

## Methods

### Immunofluorometric detection of anti- α-Gal/cetuximab IgE antibodies

Immunofluorometric α-Gal IgE detection (ImmunoCap o215, Thermo-Fisher Scientific/Phadia AB, Uppsala, Sweden) was performed according to the manufacturer’s recommendations on frozen sera (-80 °C) thawed at room temperature. Data were measured using a Phadia 250 analyzer. The positivity threshold set by the manufacturer > 0.1 kU_A_/L and the commonly accepted threshold for sensitization ≥ 0.35 kU_A_/L were used.

### ELISA detection of anti-cetuximab IgE antibodies

ELISA results were retrieved from previous studies [11, 17] with a positivity threshold of > 29 EAU (anti-cetuximab IgE Arbitrary Units).

### Cetuximab specificity of anti α-Gal IgE detection by FEIA

To study the specificity of the test towards cetuximab, inhibition tests were performed on 10 sera from the retrospective cohort by adding 20µL of cetuximab (Erbitux®, Merck KgaA, Darmstadt, Germany) or rituximab (Mabthera®, Hoffmann-La Roche, Basel, Switzerland) at 5 mg/ml in 180µL of serum.

### Patients

**Fig. 1:**
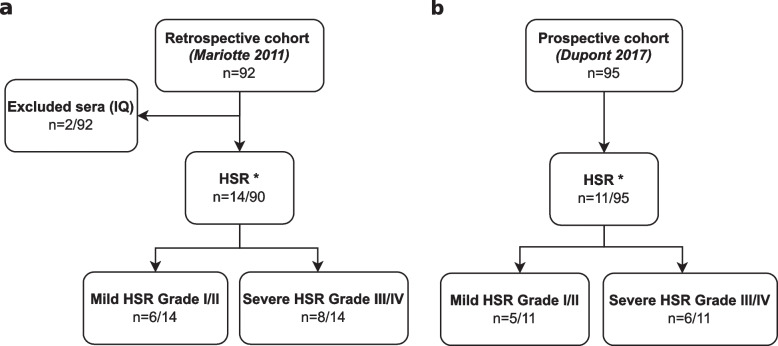
Flowchart of the study. **a** Retrospective cohort samples were taken from Mariotte D. 2011 study [17]. **b** Prospective cohort samples were taken from Dupont B.2017 study [11]. A randomized selection of 95 out of 247 patients was performed. Severity grades refer to Ring and Messmer scale [18]. Abbreviations: HSR, Hypersensitivity Reaction; IQ, Insufficient Quantity. * Anaphylactic hypersensitivity reaction based on clinical criteria

Patients included in the retrospective cohort had been previously tested in Mariotte 2011’s study [17]. Of the 92 patients, 2 had been excluded for insufficient quantity of serum. These 2 patients had not experienced HSR. Among the 90 patients left, 14 experienced an immediate HSR, including 8 severe reactions (Fig. 1A). Immediate HSR grading had been performed according to Ring and Messmer scale and severe reactions had been defined as grade III or IV [18]. The anaphylactic mechanism of the reaction had been confirmed for 6 of 7 patients for whom measurement of tryptase and histamine concentrations had been performed (Table 1).

**Table 1:** Results and characteristics of the 14 patients with HSR from the retrospective cohort and the 11 patients from the prospective cohort. In bold, positive results for each test

**Case**	**Gender**	**Age** (years)	**HSR delay after cetuximab infusion** (min)	**Severity grade **^**a**^	**Increased tryptase**^**b**^** and/or histamine**	**ELISA **(cut-off > 29 EAU)	**FEIA **(cut-off ≥ 0.35 kU_A_/L)
**Retrospective cohort results**							
P1	M	57	15	1	ND	**147**	< 0.1
P2	F	43	15	1	ND	**80**	< 0.1
P3	M	58	ND	2	no	3	**0.52**
P4	M	60	40	2	yes	**45**	**0.72**
P5	F	54	ND	2	ND	5	< 0.1
P6	M	79	20	2	ND	16	< 0.1
P7	M	64	5	3	yes	**70**	**0.53**
P8	F	54	5	3	yes	**42**	**0.73**
P9	M	59	5	3	yes	**105**	**1.12**
P10	M	53	25	3	yes	15	**1.80**
P11	M	62	15	3	yes	**31**	**3.34**
P12	M	64	10	3	ND	**60**	< 0.1
P13	M	60	10	4	ND	**40**	**2.40**
P14	M	69	5	4	ND	**> 3000**	**22.6**
**Prospective cohort results**							
P15	M	73	35	1	ND	9	< 0.1
P16	M	55	25	2	ND	2.5	**0.53**
P17	M	74	15	2	ND	**39**	0.23
P18	M	48	15	2	yes	**35**	< 0.1
P19	M	65	30	2	yes	4	< 0.1
P20	F	60	15	3	yes	**134**	**6.93**
P21	M	50	15	3	ND	**490**	**3.53**
P22	M	56	20	3	no	**55**	0.23
P23	M	68	40	3	yes	15	< 0.1
P24	F	81	ND	3	no	< 3	< 0.1
P25	M	81	< 1	4	ND	**480**	**5.63**

*Abbreviations:* *EAU* IgE Arbitrary Units, *ELISA* Enzyme Linked Immunosorbent Assay, *F* Female, *FEIA* Fluoroenzyme-Immunoassy, *HSR* Hypersensitivity reaction, *M* Male, *min* minutes, *ND* Not determined, *P* Patient

^a^ Ring and Messmer scale [18].

^b^ Increased tryptase defined as ≥ 1.2 basal value + 2 µg.L^-1^ [19].

Patients included in the prospective cohort had been previously tested in Dupont 2017’s multicentric study [11]. From 301 patients tested for anti-cetuximab IgE with ELISA, 247 have received cetuximab. Of these 247 patients, a randomized selection of 95 patients for FEIA testing has been performed. Of the 95 patients, 11 experienced an immediate HSR including 6 severe reactions (Fig. 1B). To compare data homogeneously from the two cohorts, immediate HSR grading has been recalculated according to the Ring and Messmer scale, with severe reactions defined as grade III or IV.

### Analytical parameters calculation

Sensitivity (Se), specificity (Sp), positive predictive value (PPV) and negative predictive value (NPV) were calculated for FEIA. Positive likelihood ratio (pLR), negative likelihood ratio (nLR) and positive and negative post-tests for FEIA and ELISA were calculated in the severe HSR group of the retrospective cohort.

A likelihood ratio over 10 was considered significant.

### Statistical analysis

Statistical analysis was performed using Graph Pad Prism software (Graph Pad Prism software, Inc., San Diego, CA, USA) and R software. A value of *p* < 0.05 was considered statistically significant.

Inhibition test data significance has been evaluated with the non-parametric Kruskal–Wallis test with Dunn’s multiple comparisons post-hoc test for paired series.

FEIA and ELISA discrepancies analysis was performed using Mac Nemar’s test.

A Receiver Operating Characteristic (ROC) curve analysis was performed from sensitivity and specificity results obtained from the severe HSR group of the retrospective cohort. A Youden index was calculated and  was used as a cut-off providing best performances.

## Results

### Specificity of anti-cetuximab α-Gal IgE by FEIA

**Fig. 2:**
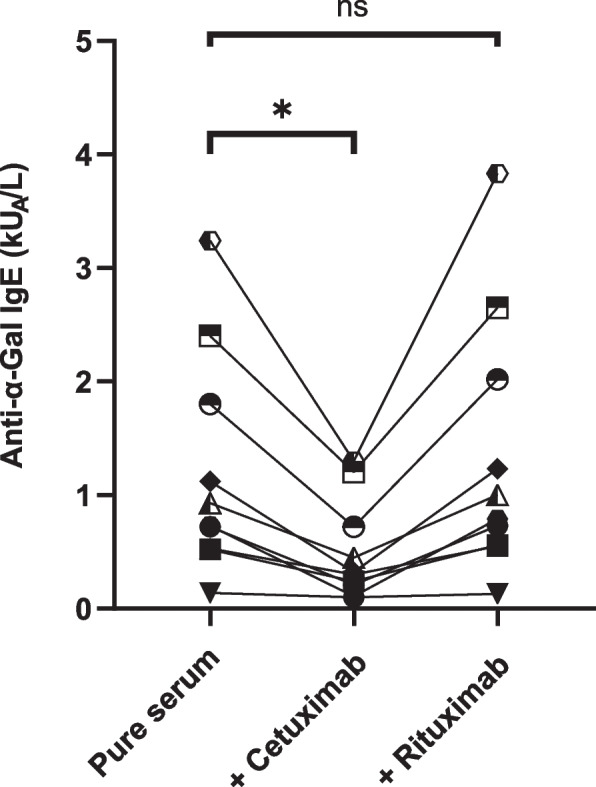
Specificity of anti-α-Gal IgE fluoroenzyme-immunoassay. FEIA ImmunoCap o215 tests were performed on 10 patients’ sera with or without Cetuximab or Rituximab addition. Each symbol represents a single patient. * *p* < 0.05; ns, non-significant

To control the specificity of the assay towards cetuximab, an inhibition test using 10 FEIA positive sera was performed with cetuximab or rituximab, a monoclonal antibody bearing the same isotype. Sera incubated with cetuximab were all significantly inhibited (*p* < 0.05) while those incubated with rituximab were not (*p* = 0.07) confirming that α-Gal specific assay was able to detect anti-cetuximab specific IgE and no other isotype specificities (Fig. 2).

### Comparison between FEIA and reference ELISA

**Fig. 3:**
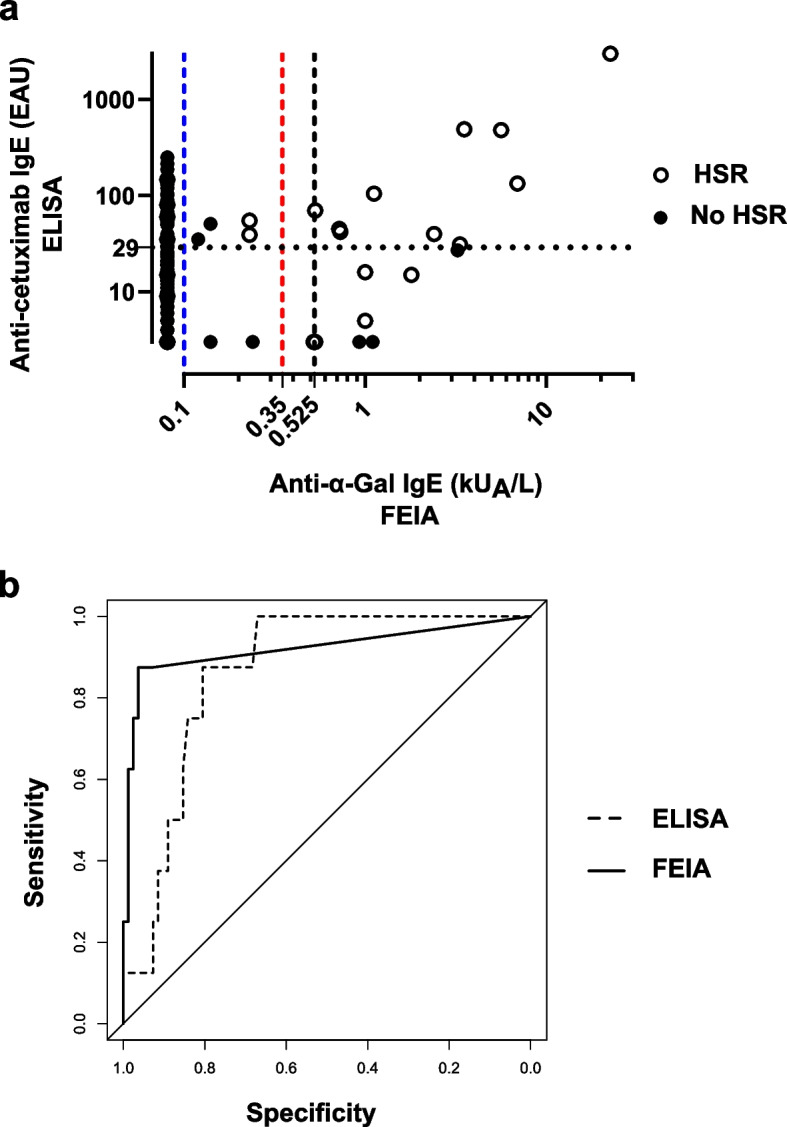
Comparison of anti-α-Gal IgE FEIA and anti-cetuximab IgE ELISA. **a** Results distribution for both cohorts combined (n = 185). For ELISA, in dotted line, 29 EAU threshold defined in Mariotte 2011. For FEIA, in dashed line, 0.1 kU_A_/L manufacturer threshold (blue), 0.35 kU_A_/L commonly defined threshold for sensitization (red) and 0.525 kU_A_/L threshold defined by a ROC curve analysis (black). **b** ROC curve analysis of retrospective cohort results. Comparison between anti-cetuximab IgE ELISA in dashed line (AUC = 0.864) and FEIA in full line (AUC = 0.921) (*p* = 0.496). Abbreviations: AUC, Area Under the Curve; ELISA, Enzyme Linked Immuno-Sorbent Assay; FEIA, Fluoroenzyme-Immunoassay; ROC, Receiver Operating Characteristic

Both cohorts combined, FEIA was performed using 185 patients’ sera (Fig. 3A). Twenty-five of them experienced an immediate HSR, among which 14 were severe (Table 1). Using the commonly accepted threshold for sensitization (≥ 0.35 kU_A_/L), FEIA was positive for 16 patients with a median of 1.46 kU_A_/L [0.52 – 22.6 kU_A_/L]. In our previous studies with the ELISA, in both cohorts, 43 patients were positive with a median of 60 EAU [30 – 3000 EAU]. For FEIA, 3 false positive results were found and 12 false negative as compared to 27 false positive results and 9 false negative for ELISA.

Overall, the rate of discordant results between ELISA and FEIA was 21.1% (39 out of 185 patients). According to Mac Nemar’s test, these discrepancies were statistically significant (*p* < 0.0001). Among these discrepancies, 27 out of 39 (69.2%) were due to false-positive and 3 to false-negative (7.7%) with ELISA. 3 false-positive (7.7%) and 6 false-negative (15.4%) were observed with FEIA.

Importantly, of the 14 patients with severe reactions, FEIA and ELISA tests were both positive for 9 patients, both false-negative for 2 patients and discordant for 3 patients: one false-negative result with ELISA (patient 10) and two false-negative results with FEIA (patient 12 and 22). The immediate-type hypersensitivity mechanism of the reaction was confirmed for patient 10. There was no significant tryptase or histamine release for patient 22 questioning the allergic nature of the reaction and the false-negative FEIA status. For patient 12, mediators release was not determined, and the nature of the reaction cannot be ascertained.

### Evaluation of FEIA diagnostic performances using the retrospective cohort

According to the design of Dupont 2017’s prospective study, patients with high levels of anti-cetuximab IgE may have been rejected for cetuximab treatment after a multidisciplinary oncology expert meeting. Therefore, the calculation of performances could only be performed using the retrospective cohort.

Analytical performances for severe reactions (grade III or IV) diagnosis were first calculated using the manufacturer technical cut-off (> 0.1 kU_A_/L). They were comparable for FEIA and ELISA techniques for sensitivity (FEIA: 87.5% vs ELISA: 87.5%) and NPV (FEIA: 98.7% vs ELISA: 98.5%) but the specificity (FEIA: 93.9% vs ELISA: 82.1%) and PPV (FEIA: 58.3% vs ELISA: 33.3%) were higher for the FEIA test (Table 2).

**Table 2:** Analytical performances of anti-cetuximab IgE detection by ELISA or FEIA in the severe HSR group of the retrospective cohort. In bold, likelihood ratios over 10 which were considered significant

	**ELISA** Mariotte D. et al*.* 2011 [17]	**FEIA** ImmunoCAP o215		
Threshold	> 29 EAU	> 0.1 kU_A_/L (defined by the manufacturer)	≥ 0.35 kU_A_/L (commonly accepted threshold for sensitization)	≥ 0.525 kU_A_/L (defined by the ROC curve)
Se (%)	87.5	87.5	87.5	87.5
Sp (%)	82.1	93.9	95.1	96.3
PPV (%)	33.3	58.3	63.6	70.0
NPV (%)	98.5	98.7	98.7	98.8
pLR	4.89^a^	**14.4**	**17.9**	**23.9**
nLR	0.15^a^	0.13	0.13	0.13
pPP (%)	19.6^a^	44.0	49.6	56.7
nPP (%)	0.80^a^	0.70	0.70	0.70

*Abbreviations:* *EAU* IgE arbitrary units, *nLR* Negative likelihood ratio, *NPV* Negative predictive value, *nPP* negative post-test probability, *pLR* Positive likelihood ratio, *pPP* Positive post-test probability, *PPV* Positive predictive value, *Se* Sensitivity, *Sp* Specificity

^a^ Parameters not calculated in Mariotte 2011 study

Performances were also calculated using the commonly accepted threshold for sensitization (≥ 0.35 kU_A_/L) showing an increase in specificity (95.1%) and PPV (63.6%) as compared to the manufacturer threshold (Table 2).

The ROC curve analysis (Fig. 3B) showed a minimal and non-significant improvement of the area under the curve (AUC) equal to 0.921 for the FEIA test as compared to 0.864 for the ELISA test (*p* = 0.496). The calculated Youden index defined a threshold of ≥ 0.525 kU_A_/L, improving FEIA performances in specificity (96.3%) and PPV (70.0%) as compared to > 0.1 kU_A_/L and ≥ 0.35 kU_A_/L thresholds (Table 2).

For ELISA and FEIA at all thresholds, negative likelihood ratios (nLR) were not very powerful and below 1. In contrast, while ELISA positive likelihood ratio (pLR) was 4.89, FEIA pLRs were over 10 with > 0.1 kU_A_/L, ≥ 0.35 kU_A_/L and ≥ 0.525 kU_A_/L thresholds (14.4, 17.9 and 23.9 respectively) (Table 2).

Based on the 5.2% prevalence in our population of severe immediate HSR [17], post-test probabilities have been calculated. Using the manufacturer detection cut-off (> 0.1 kU_A_/L) for the FEIA test, the negative post-test probabilities of severe HSR for both techniques were comparable (FEIA: 0.7% vs ELISA: 0.8%) while the positive post-test probability was higher for FEIA (FEIA: 44.0% vs ELISA: 19.6%). It reached 49.6% with the ≥ 0.35 kU_A_/L threshold and 56.7% with the ≥ 0.525 kU_A_/L threshold while the negative post-test probability remained at 0.7% (Table 2).

## Discussion

This study showed that the FEIA technique has high performances in detecting α-Gal epitopes of cetuximab and performed better for HSR risk assessment than the ELISA technique.

FEIA based techniques have first been developed using homemade or research ImmunoCaps coated with cetuximab [1, 20]. Nevertheless, the standardized commercial reagent then provided was an ImmunoCap coated with bovine thyroglobulin, an α-Gal-rich protein. The availability of this automated method using a clinically validated reagent used in the diagnostic of α-Gal-dependent meat allergy led us to evaluate the correlation between this FEIA ImmunoCap o215 and our ELISA method for the screening of patients at risk of immediate HSR before cetuximab infusion. For this purpose, we used collections from two previous studies based on data from more than 300 patients tested prior to cetuximab treatment among whom 185 were evaluated with FEIA [11, 17].

The specificity of the FEIA test for cetuximab as compared to another IgG1 kappa antibody confirmed its relevance in detecting cetuximab sensitization and its interest for the screening of patients reactivity before treatment.

Discrepancies between ELISA and FEIA were numerous (21.1%) and statistically significant (*p* < 0.0001). However, most of them were related to false positive results in ELISA, in agreement with the lower specificity of this technique. FEIA showed a better discrimination of positive patients and a comparable discrimination of negative patients.

Intrinsic differences between the two techniques could explain the observed discrepancies. First, the ELISA technique used cetuximab as the antigen phase while the FEIA technique used bovine thyroglobulin. This can result in modified molecular interactions, with extra epitopes recognized, although our control of specificity confirmed an interaction with α-Gal with FEIA. In addition, the thyroglobulin had been chosen for its high content in α-Gal epitopes, which added to the ImmunoCap format might improve the capture. Some other analytical parameters such as serum incubation time and detection method varied depending on the technique. Moreover, the determination of inter-assay and intra-assay coefficients of variation of ELISA and FEIA for IgM and IgG antibody assays has shown that ELISA was less accurate for low values (no data were available for IgE assays) [21]. This is consistent with the high number of false positive results with ELISA observed for low values in this study.

The interpretation of the prospective cohort results was limited by the design of the study resulting in the rejection for cetuximab treatment of some patients with high levels of anti-cetuximab IgE. Consequently, calculation of the specificity and the PPV was not possible and was only performed on the retrospective cohort. The analysis of the retrospective cohort results showed equal performances between FEIA and ELISA tests for patients with severe reactions. Sensitivity and NPV were comparable for both techniques while specificity and PPV were improved with FEIA.

These results are in line with a meta-analysis performed to assess the diagnosis accuracy of anti-α-Gal IgE in predicting the risk of cetuximab immediate HSR. From a compilation of 6 studies, the authors reported a pooled sensitivity of 73% (95% CI 62–81%) and a pooled specificity of 88% (95% CI 79–94%) [13]. Two studies included in this meta-analysis used ImmunoCap o215. In a small Korean prospective cohort (*n* = 64), Park et al*.*, using the commonly accepted ImmunoCap threshold of 0.35 kU_A_/L, described a sensitivity, specificity, PPV and NPV of 100%, and Weiss et al*.* calculated a NPV of 100% in a 60-patient retrospective cohort with a threshold set at > 0.1 kU_A_/L [22, 23]. In Park et al*.* study, ELISA and FEIA provided equal performances, results were positive in both tests for the 4 HSR patients and negative for the 60 other patients.

Likelihood ratios are parameters independent of the population prevalence. The ELISA test did not confirm the higher risk of hypersensitivity reaction to cetuximab. Indeed, the pLR was 4.89 and not enough to make the diagnosis likely. In contrast, FEIA was more discriminating with pLRs over 10, regardless the threshold used from 14.4 with the > 0.1 kU_A_/L cut-off and reaching 23.9 with the ≥ 0.525 kU_A_/L cut-off.

The positive post-test probability calculation in our population was nearly 20% of risk of severe HSR for ELISA and increased from almost 45% (> 0.1 kU_A_/L cut-off) to almost 60% (≥ 0.525 kU_A_/L cut-off) with FEIA.

Whatever the methods and thresholds used, nLR were between 0.13 and 0.15. Thus, in case of a negative result, there is still a very low residual risk of undergoing a severe reaction (below 1% in our population). Accordingly, a negative result should not allow to avoid an appropriate monitoring when treatment is initiated, although it might be lighter.

If calculated, the sensitivity in the prospective cohort for severe HSR patients would be lower than that found in the retrospective cohort (FEIA vs ELISA: 66.7 vs 87.5%, at > 0.1 kU_A_/L threshold and 50.0 vs 87.5%, at ≥ 0.35 and ≥ 0.525 kU_A_/L thresholds) in keeping with the lower number of false positive results. The NPV would be equivalent, almost 98% whatever the technique or thresholds.

This FEIA test is a commercial test available everywhere. Therefore, it can be easily used to screen all patients eligible for cetuximab treatment and contribute to avoid immediate HSR. The screening of anti-α-Gal IgE is consistent with the development of personalized medicine. It follows the same strategy as the pre-therapeutic detection of dihydropyrimidine dehydrogenase deficiency prior starting 5-Fluorouracil infusions to prevent severe drug toxicities [24].

Sensitization to the α-Gal epitope as detected by anti-α-Gal IgE positivity is not, as for any allergen, synonymous with allergy but indicates a higher risk of reaction that should require a careful monitoring at the beginning of treatment. On the other hand, a negative test can facilitate the management of patients although never eliminating completely a risk of reaction.

Altogether, our results comfort previous studies on α-Gal IgE detection and suggest that assessing patient's HSR risk based on FEIA ImmunoCap o215 test positivity can improve the selection of cetuximab therapy for better outcome and prognosis. In addition to confirming the interest of FEIA, our study based on a ROC analysis suggests a threshold of ≥ 0.525 kU_A_/L. Although this threshold would require further consolidation, it is obvious that it will be difficult to refine it owing to the now well-known risk of reaction in α-Gal sensitized patients.

## Conclusion

In this study, the FEIA ImmunoCAP o215 test characteristics were evaluated for anti-α-Gal / anti-cetuximab IgE screening before cetuximab treatment. We confirmed that this FEIA method is adapted for the detection of anti-α-Gal / cetuximab IgE antibodies and to identify patients at high risk of severe immediate-type HSR. The analytical performances for FEIA are better than those of our previously published anti-cetuximab IgE ELISA. The use of a positivity threshold ≥ 0.525 kU_A_/L defined by ROC analysis or the classical ≥ 0.35 kU_A_/L, allows the calculation of likelihood ratios indicating a more significant risk of severe immediate reactions in case of a positive FEIA test and a lower risk in case of a negative one. The implementation of this standardized and automated assay available in many laboratories could help decreasing cetuximab-induced anaphylaxis and adapting the monitoring of patients during the first injection.

## Data Availability

The datasets used and/or analysed during the current study are available from the corresponding author on reasonable request.

## Abbreviations

α-Gal: Galactose-alpha-1,3-galactose
AUC: Area under the curve
EAU: Anti-cetuximab IgE arbitrary units
EGFR: Epidermal growth factor receptor
ELISA: Enzyme-linked immunosorbent assay
FEIA: Fluoroenzyme-immunoassay
HSR: Hypersensitivity reaction
nLR: Positive likelihood ratio
NPV: Negative predictive value
pLR: Positive likelihood ratio
PPV: Positive predictive value
ROC: Receiver operating characteristic
Se: Sensitivity
Sp: Specificity
